# Microbial Transformations of Organically Fermented Foods

**DOI:** 10.3390/metabo9080165

**Published:** 2019-08-10

**Authors:** Ruma Raghuvanshi, Allyssa G. Grayson, Isabella Schena, Onyebuchi Amanze, Kezia Suwintono, Robert A. Quinn

**Affiliations:** Department of Biochemistry and Molecular Biology, Michigan State University, East Lansing, MI 48824, USA

**Keywords:** Fermented Food, metabolomics, GNPS, molecular networking, microbiome

## Abstract

Fermenting food is an ancient form of preservation ingrained many in human societies around the world. Westernized diets have moved away from such practices, but even in these cultures, fermented foods are seeing a resurgent interested due to their believed health benefits. Here, we analyze the microbiome and metabolome of organically fermented vegetables, using a salt brine, which is a common ‘at-home’ method of food fermentation. We found that the natural microbial fermentation had a strong effect on the food metabolites, where all four foods (beet, carrot, peppers and radishes) changed through time, with a peak in molecular diversity after 2–3 days and a decrease in diversity during the final stages of the 4-day process. The microbiome of all foods showed a stark transition from one that resembled a soil community to one dominated by Enterobacteriaceae, such as *Erwinia* spp., within a single day of fermentation and increasing amounts of Lactobacillales through the fermentation process. With particular attention to plant natural products, we observed significant transformations of polyphenols, triterpenoids and anthocyanins, but the degree of this metabolism depended on the food type. Beets, radishes and peppers saw an increase in the abundance of these compounds as the fermentation proceeded, but carrots saw a decrease through time. This study showed that organically fermenting vegetables markedly changed their chemistry and microbiology but resulted in high abundance of Enterobacteriaceae which are not normally considered as probiotics. The release of beneficial plant specialized metabolites was observed, but this depended on the fermented vegetable.

## 1. Introduction

Fermented foods have been part of the human diet for millennia [1,2]. Though experimentation with fermented foods began with ancient societies, such as strain cultivation and propagation, more recently, a resurgence in the science of fermented foods has begun with the explosion of research into the human microbiome [3,4]. It is well established that diet directly influences the structure and function of our gut microbiome, which in turn, influences one’s overall health [5]. It has been argued that those living in a Westernized society have been developing a gut microbial dysbiosis since the industrial revolution [6]. There is a belief that consumption of probiotic products, such as fermented foods, may begin to reverse this process and these foods are becoming more recognized for their health benefits and availability to consumers. However, we still have a relatively poor understanding of the microbes and microbial processes that exist in fermented foods, particularly pertaining to the chemical transformations that occur due to natural microbial activity. These natural fermentations are becoming more popular in westernized countries with many organic farmers and organic food consumers being avid fermenters of fresh vegetables.

Many East Asians societies directly ferment vegetables with either starter cultures or naturally occurring bacteria and some of the microbial transformations in these foods have been studied. For example, the molecular and microbial changes in the fermentation of cabbage (*Brassica rapa*) into kimchi resulted in increases in the levels of lactic acid, glycerol, pyrotartaric acid, pentanedioic acid, 2-keto-1-gluconic acid, ribonic acid, isocitric acid, and palmitic acid and a general decrease in amino acids and sugars [7]. The metagenome of kimchi fermentation has also been explored enabling the identification of microbial species involved and their functional characterization. Lactic acid bacteria, such as *Leuconostoc, Lactobacillus* and *Weissella*, were the primarily genera in fermented kimchi. These organisms used heterolactic fermentation to produce mannitol, lactate, acetate, and ethanol as fermentation products [8]. Lactobacilli and yeasts were found to be the primary natural fermenters of the grain-based African fermented beverage togwa, producing small aldehydes, methyl-butanal, methy-butanols and other small fermentation products [9]. Naturally, most chemical and microbial analyses of fermented foods have focused on small molecules from microbial fermentation, particularly organic acids and other byproducts of microbial metabolism. Less studied, are the larger metabolites such as plant natural products and how are they modified by microbial fermentation. Many of these natural products are believed to have unique health benefits, which may be a hidden source of benefit from these fermented foods.

This study used liquid chromatography-tandem mass spectrometry (LC-MS/MS), molecular networking on the Global Natural Products Social molecular networking (GNPS) infrastructure, and 16S rRNA gene sequencing to explore the microbial transformations of four organically fermented vegetables. We paid particular attention to flavonoids and other plant natural products released from these foods, to determine whether natural microbial food fermentation may make these compounds more bioavailable by releasing them from the plant prior to digestion.

## 2. Methods

### 2.1. Sample Collection

The carrots (*Daucus carota*), beets (*Beta vulgaris*), peppers (*Capsicum annuum*), and radishes (*Raphanus raphanistrum*) were purchased from the Michigan State University Student Organic Farm (East Lansing, MI, USA) Stand and stored at 4 °C for 48 h. Each vegetable was chopped into smaller pieces and four 0.5g samples of each were collected and stored at −20°C prior to fermentation. Thus, a replicate represented pieces of one individual vegetable from the batch purchased on the same day from the MSU organic farm. For each vegetable, four 478 mL (one pint) jars were filled ¾ with the chopped vegetables and enough salt water (50 g/L, FisherSci NaCl) to cover them was added. One jar of each vegetable type was autoclaved for 30 min as a control (day 0 sample was taken prior to autoclaving) and the other three replicates used for fermentation with no prior sterilization. All of the jars were sealed and left at room temperature for 4 days (96 h) to ferment. At 24 h intervals after the first sampling, 500 μL of liquid from each jar was placed into an microcentrifuge tube and filled to 1 mL with the corresponding chopped vegetable. Samples were taken every 24 h both for microbiome and metabolome analysis and stored at −20 °C until all the samples were collected and then moved to −80 °C (experimental schematic available in Fig. S1). The pH of each sample was measured after thawing samples immediately prior to metabolite extraction.

### 2.2. Extractions

Metabolite extractions were performed by adding 1 mL of LC-MS grade methanol to a 300 μL volume of each sample. The samples were vortexed and then extracted at 4 °C overnight. After extraction, the samples were spun to pellet particulates and the supernatant was used for mass spectrometry.

### 2.3. Mass Spectrometry

A 50 μL aliquot of each sample extract was diluted 1:3 in 50% methanol containing 2 μM phenol red standard and subjected to LC-MS/MS analysis on a Thermo^TM^ QExactive^TM^ mass spectrometer coupled to a Vanquish HPLC system. The mobile phase was an increasing gradient of solution A (100% LC-MS grade water) to solution B (100% LC-MS grade acetonitrile) both containing 0.1% formic acid. The stationary phase column was a Waters^®^ Acquity^®^(Wood Dale, IL, USA) UPLC BEH C18 column, 2.1 mm × 100 mm. The chromatographic program was a 12-min-long run that began with a hold at 5% solution B for 30 s then an increasing linear gradient of solution B from 5–99% over 8.5 min. This 99% B solution was then held for 1.5 min followed by a switch to 0% B for the remaining 1.5 min. The full MS^1^ mass spectra were collected in positive mode at a resolution of 17,500, an automatic gain control (AGC) target of 5e5, scan range of 150 to 1500 m/z for the full MS mode (minutes 2–10 of run). The MS/MS spectra were collected at the same resolution and AGC target with an isolation window of 1.0 *m/z*, a loop count of 5 and stepping of energies 20, 30 and 40 NCE. The data dependent settings included a dynamic exclusion of 1.0 s.

### 2.4. Metabolome Data Analysis

Thermo^TM^ QExactive^TM^ .raw files were first converted to the centroided .mzXML file format and uploaded to the GNPS database and MassIVE data repository under MassIVE ID MSV000084067. Molecular networks were generated on GNPS with the following parameters: parent mass tolerance of 0.03 Da, fragment mass tolerance of 0.03 Da, 0.65 minimum cosine score, 4 minimum matched peaks, and minimum cluster size of 2. Library search parameters were the same as the networks. Feature finding was performed with the mzMine v. 2.0 software with the following parameters: MS^1^ noise tolerance of 500,000, MS^2^ noise tolerance of 50,000, and retention time tolerance of 0.2 min. The chromatograms were deconvoluted, deisotoped, aligned with a retention time tolerance of 0.2 min, filtered such that a peak must be found in at least 3 samples, and the missing values were imputed using gap-filling for subsequent statistical analysis. The Thermo^TM^ Compound Discoverer^TM^ software v3.0.0.294 was also used to identify and quantify known compounds in the untargeted metabolomics dataset. The ‘untargeted metabolomics workflow’ default parameters were used to identify knowns with mzCloud MS/MS searching and quantification of feature abundances. The samples were grouped by vegetable type and day of fermentation for analysis.

### 2.5. Microbiome Sequencing

The microbiome data was generated according to the protocols developed from the Earth Microbiome Project with some deviations described below [10] (http://www.earthmicrobiome.org/protocols-and-standards/). Briefly, genomic DNA was extracted from the combined food and brine samples of each fermented food using the Qiagen^®^ PowerSoil^®^ (Hilden, Germany) genomic DNA extraction kit as described in its protocol. Sequencing was performed using Illumina MiSeq available at Michigan State University genomics core facility. Briefly, using dual indexed Illumina compatible PCR primers 515f/806r targeted amplicon libraries of the microbial 16S rRNA V4 hypervariable region were constructed following the methods of [11]. The PCR amplicons were batch normalized using Invitrogen SequalPrep DNA Normalization Plates and the product was pooled. The pool was QC’d and quantified using a combination of Qubit dsDNA HS, Agilent 4200 TapeStation HS DNA1000 (Wood Dale, IL, USA) and Kapa Illumina Library Quantification qPCR assays (San Diego, CA, USA). The pool was loaded onto a Standard Illumina MiSeq v2 flow cell and sequencing was performed in a 2 × 250bp paired end format using a MiSeq v2 500 cycle reagent cartridge. Complementary to the 515f/806r sequences custom sequencing and index primers were added to appropriate wells of the reagent cartridge. Illumina Real Time Analysis (RTA) v1.18.54 was used for base calling and its output was demultiplexed and converted to FastQ format with Illumina Bcl2fastq v2.19.1. Finally, the data was processed and analyzed using the Qiita [12] software with the Greengenes database. Alpha- (Shannon index) and beta-diversity (weighted UniFrac) analysis were calculated using data analyzed with the Deblur algorithm [13] and taxonomy assignments were performed with the OTU clustering method (97% identity).

### 2.6. Statistical Analysis

Beta-diversity of the metabolomic data was calculated using the Bray-Curtis dissimilarity and visualized in Principle coordinate analysis space (PCoA) using the EMPeror software [14]. Changes in the metabolome through time were analyzed using a random forest (RF) linear regression approach with the randomForest package in the *R* statistical software. The percent of data explained by the time of fermentation was used to assess how the fermentation progressed through time and metabolites that changed most significantly through the fermentation were identified using variable importance plots of this RF regression.

## 3. Results

### 3.1. Overall Metabolome Changes in Organic Fermented Foods

**Figure 1:**
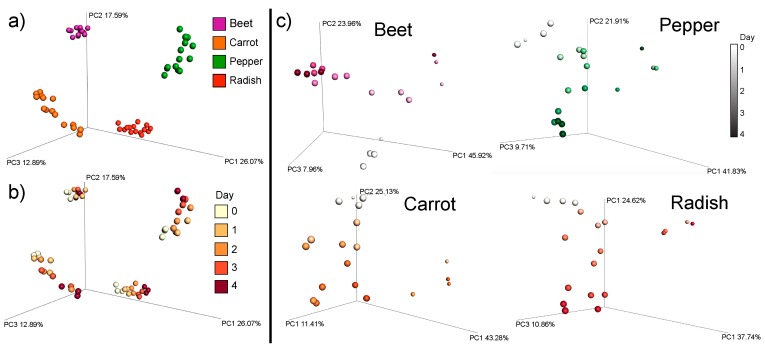
Principle coordinate analysis of a Bray-Curtis distance matrix of metabolomic data relationships among fermented food samples through time. The overall data relationship based on (**a**) different fermented food type and (**b**) days of fermentation. (**c**) The isolated analysis of each food as it is fermented for the 4-day time frame. Samples are colored as a spectrum from white to dark color corresponding to time for each vegetable. The autoclaved samples in this panel are smaller spheres.

**Figure 2:**
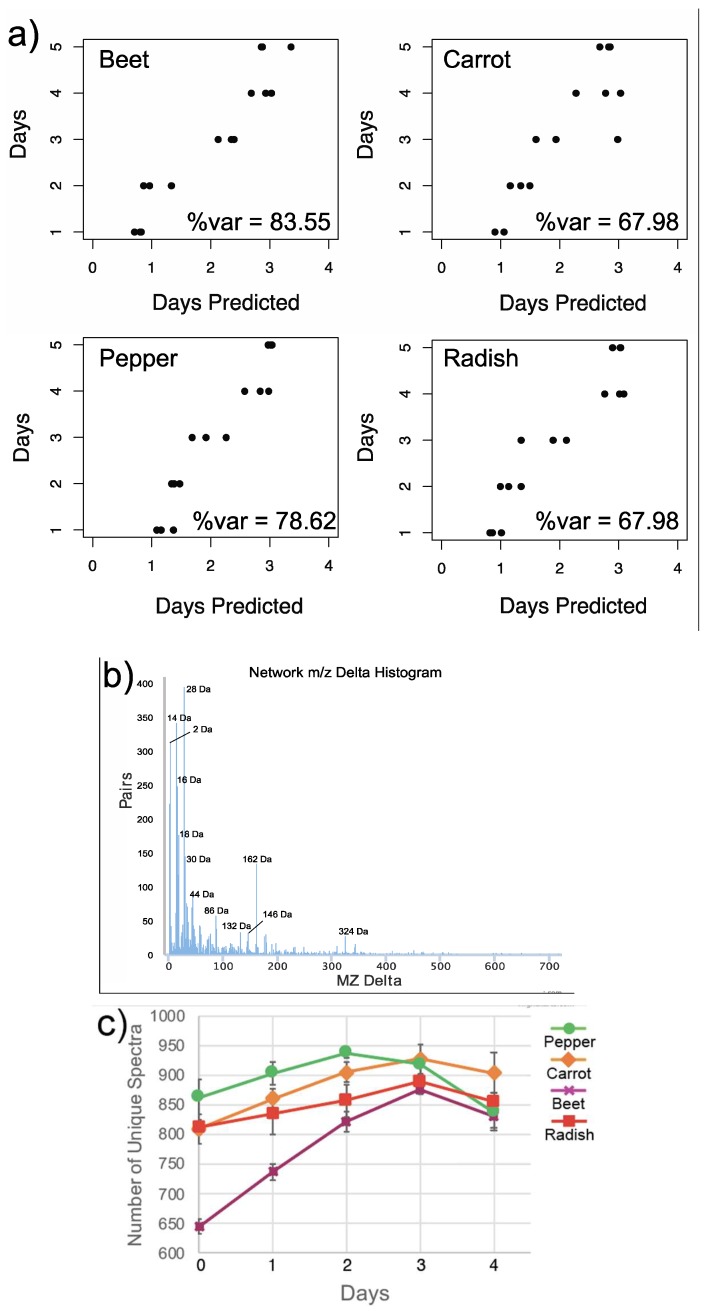
(**a**) Scatterplot of actual vs. predicted days of fermentation from RF regression for each food type. The RF machine learning algorithm predicts the assignment of the sample in days based on the trend in the metabolomic data. The percent variance explained by days in the metabolomic data is also shown. (**b**) The most common transformations across dataset based on molecular networking in GNPS plotted as the pairs (represented by one networking edge and difference in *m/z* of each pair. The abundance of each edge represents its occurrence in the network between two MS/MS pairs). (**c**) Unique MS/MS spectral richness through the fermentation based on molecular networking in GNPS and the standard deviations of the replicates.

The autoclaved samples had different metabolomic signature than the conventionally fermented samples and changed comparatively little through time (Figure S2). The metabolome of the fermented food samples separated primarily based on food type (Figure 1a), reflecting the different chemical signatures of the different plant species. Fermentation of the foods for four days induced large changes in the metabolomic signature (Figure 1b,c). An RF linear regression was used to assess how the metabolomic data reflected changes in the food samples through time (Figure 2a). The percent of variance explained by time in days from the RF was used as a measure of the degree of change in the fermented food metabolome. The autoclaved samples had no linear relationship with time with the RF regression indicating no progressive changes were occurring in the metabolome (Table S1). All conventionally treated food types however had strong metabolite changes through the four-day fermentation with beet having the largest changes (83.55% variance explained), followed by pepper (78.62% variance explained) and then carrot and radish (67.98% variance explained). All vegetables displayed a progressive linear change in the metabolomic data except for day 4, where all samples were incorrectly classified as day 3. This indicates that the metabolomic data ceased its linear progression of change after day four, likely due to the fermentation process reaching an endpoint or a stationary phase.

We used molecular networking on the GNPS platform (gnps.ucsd.edu) to identify unique MS/MS spectra in the database (putative molecules) and their relationships. By quantifying the mass differences between nodes with connected edges we are able to identify the most common transformations across the dataset [15]. As expected, the most common mass shifts represent 2 Da (H_2_), 14 Da (CH_2_), 16 Da (OH), 18 Da (H_2_O), but we also observed some less common biochemical transformations such as 30 Da, 44 Da, and some larger mass deltas such as 132 Da, 146 Da, 162 Da, and 324 Da (Figure 2b). These larger mass deltas corresponded to the loss of sugar moieties on different molecules. A loss of 132 m/z Da represents a pentose sugar, 142 m/z a rhamnose sugar, 162 m/z a hexose sugar, and 324 m/z a dihexose.

There were 5392 unique spectra (putatively molecules) identified according to the molecular networking MS cluster algorithm. There were strong metabolite changes in the fermented foods through time seen as an initial increase in metabolite richness. All vegetables had a significant increase in metabolite richness through time except for peppers (Table S2). There was also an observed decrease at days 2 or 3 depending on the vegetable (Figure 2c). Beats had the largest increase in metabolite diversity starting at 644 unique spectra and peaking at 876 on day 3, peppers had the largest decrease in metabolite richness where there was a drop in 100 unique spectra detected between days 2 and 4.

The changes in pH in the brine of each fermented food were also measured. The starting pH of the brine depended on the vegetable, with beets being highest and pepper lowest (Figure S3). Through the fermentation all the samples decreased in pH but ended up at approximately the same level at day 4 (mean pH of all vegetables = 3.79, SD = 0.25). This meant that the beets had the largest change in pH moving over 3 units, whereas peppers had little change moving less than 1 unit over the four-day fermentation (Figure S3).

### 3.2. Microbiome Dynamics

**Figure 3:**
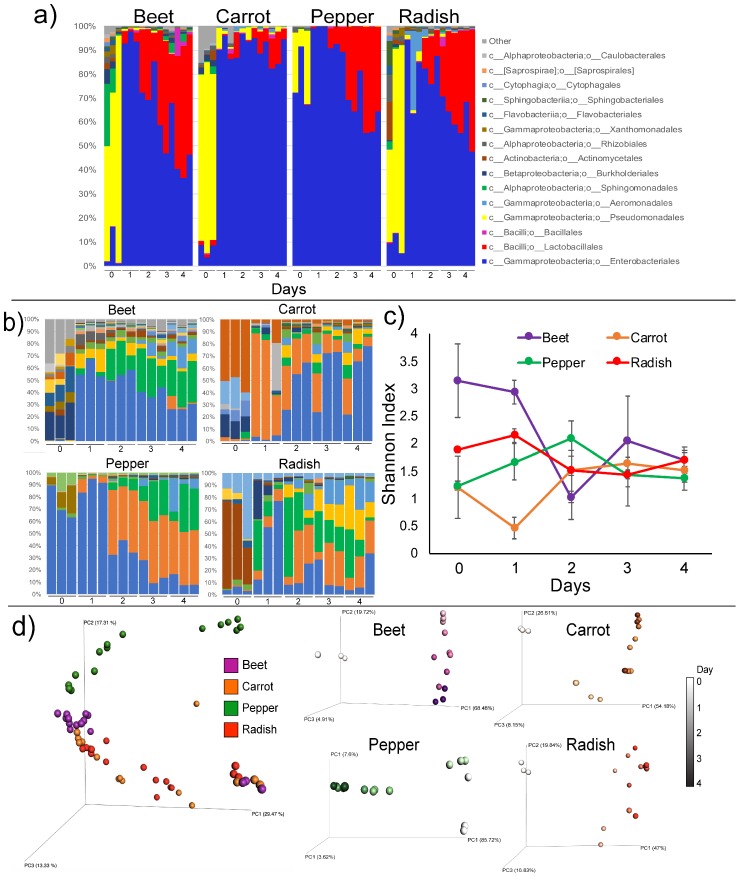
Microbiome changes in the organically fermented foods. (**a**) Taxa changes at the Order level in all fermented food through time, each of the three replicates is shown as its own bar in the bar graph. (**b**) Taxa changes at the OTU level in different fermented foods through time. (**c**) Shannon index of microbial diversity changes in each fermented food through time. (**d**) Principle coordinate analysis of the weighted UniFrac [16] distance of the Deblurred data for the whole microbiome dataset and each individual vegetable. In the PCoA plots of each individual vegetable, time is denoted as darkening of color for each representative vegetable according to their representative color scale.

Sequencing of 16S rRNA gene amplicons was used to assess the microbial changes during the organic food fermentations. At the taxonomic level of Order, the major changes through time were a decrease in Enterobacteriales and increase in Lactobacillales along with changes in some lower abundance taxa (Figure 3a). Interestingly, the microbiome of the fermented foods before fermentation were dominated by the order Pseudomonadales, which was rapidly depleted within the first day of fermentation. Analysis at a lower taxonomic level revealed that changes in microbial diversity depended on the food type (Figure 3b, Figures S4–S7). Alpha-diversity of the microbiome data did not follow a notable pattern, except in beets where a noted reduction in alpha-diversity occurred (Figure 3c). Each fermented food had a unique microbiome at the starting point and through time, though the fermentation was dominated by particular bacterial groups (Figure 3b,d). OTUs belonging to the Enterobacteriaceae and *Erwinia* sp. dominated fermentation of all vegetables, with the same OTU present in carrot and beet. *Lactococcus* and *Leuconostoc* sp. also increased in relative abundance in the different foods through time. Prior to the fermentation pseudomonads dominated the microbiome of beet, carrot and radish. These microbes were quickly depleted during the fermentation.

### 3.3. Changes in Individual Metabolites through Time

**Figure 4:**
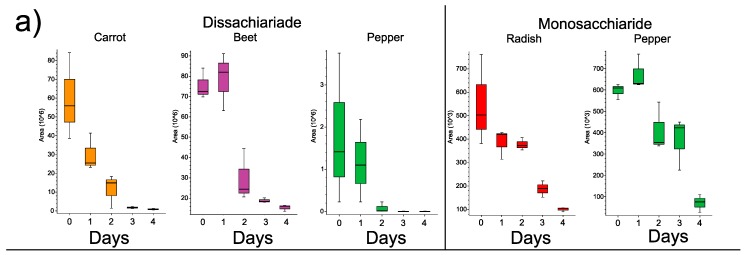

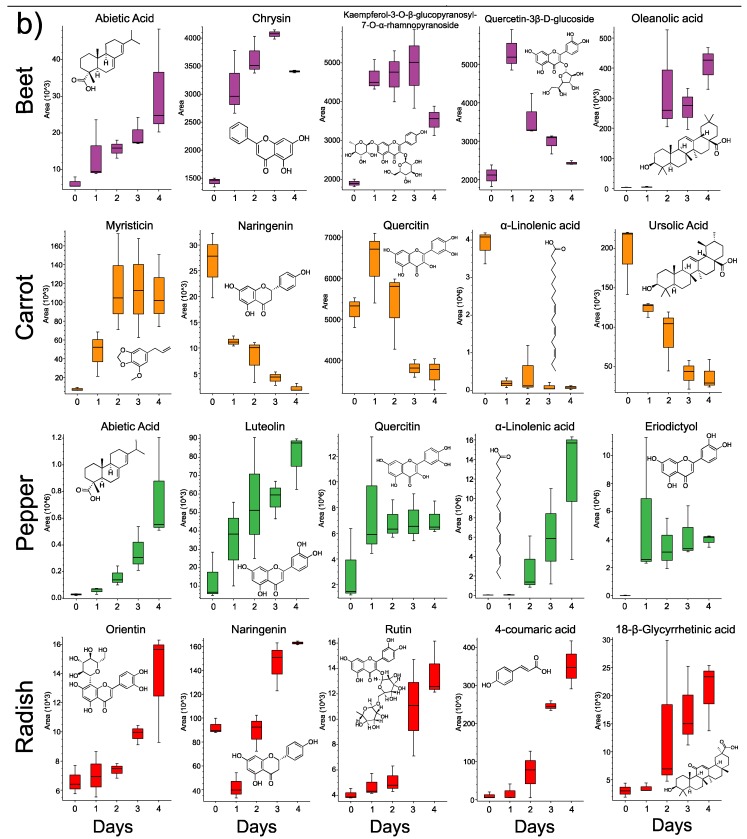
Changes of unique metabolites through the organic food fermentations. (**a**) Boxplots of changes in sugar abundance through the five-day fermentation in different vegetables. (**b**) Boxplots of changes in plant natural products detected through GNPS and Compound Discoverer library searching in the four vegetables fermented in this study. Putative structures of the compounds are also shown and the plots are ordered left to right as triterpenoids, flavonoids and other compounds for each specific vegetable.

GNPS library searching and the Thermo^TM^ Scientific Compound Discoverer^®^ Software were used to identify known MS/MS spectra in the dataset (Figure S9). All compounds in this study were identified at the level of class 2 according to the metabolomics standards initiative [17]. Sugars were depleted through time in all vegetables. Though the exact molecular structures of these compounds cannot be discerned using our MS/MS methods, a disaccharide and monosaccharide were identified as decreasing through the fermentation (Figure 4a).

Plant natural products were of particular interest to this study due to their known bioactive and potential health benefits. Flavonoids, triterpenoids and other small molecules were highly dynamic during the fermentation and unique to each vegetable, with some overlap in specific flavonoids across vegetables such as quercitin. The aglycone flavonoids chrysin, naringenin, luteolin and eriodictyol were identified as well as the glycones rutin, orientin, and glycones of kaempferol and quercitin (Figure 4b, Figure S9 level two Metabolomics Standards Initiative [17]). The changes in these molecules through the fermentation depended on the vegetable. In peppers and radishes, flavonoids increased through time, whereas in carrots there was a general decrease in these compounds. Interestingly in the beet fermentation, all flavonoids increased initially in the first few days, but then decreased in the latter stages indicating that the microbes may have been responsible for the release of these compounds from the plant and their subsequent degradation. Other plant natural products increased in abundance during fermentation including abietic acid, which rapidly increased in beets and peppers, 4-coumaric acid and 18-β-glycyrrhetinic acid in radishes, and the triterpenoids oleanolic acid in beets. Ursolic acid in carrots, much like the flavonoids, decreased in abundance through the fermentation. The fatty acid linoleic acid also increased in peppers but decreased in carrots (Figure 4b).

### 3.4. Anthocyanin Dynamics in Radishes

**Figure 5:**
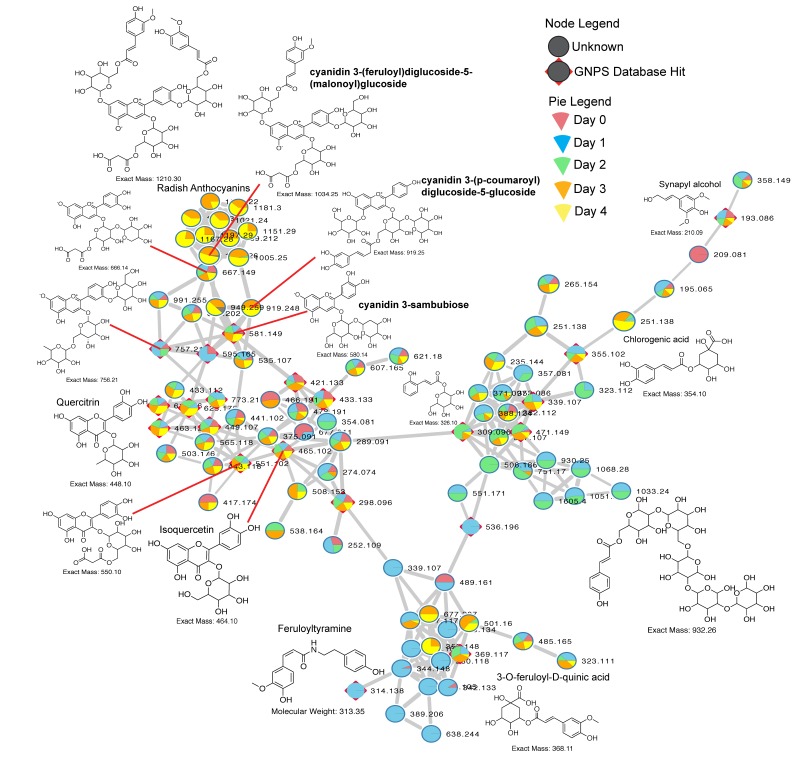
Molecular network of anthocyanins, flavonoids, ferulic acids and related molecules from the fermented food LC-MS/MS dataset (anthocyanins found only in radishes). Each node represents a uniquely clustered MS/MS spectrum and putative metabolite. Nodes are colored using pie charts which correspond to the spectral count in the different fermentation days according to the legend. Related metabolites are connected by edges according to their MS/MS cosine score and the width of the edge is determined by the score. Compounds with a GNPS database hit are highlighted using a red square behind the node. Putative structures and compounds names are shown when discernable through the literature or library matches.

In the molecular network of all metabolites we discovered a large cluster of spectra containing known flavonoids and other compounds, which were highly dynamic through the fermentation. As such, we further investigated the chemistry of these spectra and propagated annotations through the network to better understand what these spectra were representing and how the metabolites were changing through time. Many of these compounds were glycone flavanoids and anthocyanins, such as quercitin and cyanidin, which were found in higher abundance in the latter days of the food fermentation. Propagating the annotations of these complex glycone flavonoids led to the discovery of a cluster of closely related anthocyanins present in radishes (Figure 5, Figure S8). These molecules were only detected in day 3 and 4 of the fermentation of this vegetable. These anthocyanins were putatively identified by MS/MS and parent mass matching and by comparing to known compounds in the literature [17,18]. We subsequently ran a correlation analysis of one of the most abundant anthocyanins detected (*m/z*1005.248, Figure 5) and found that it was highly correlated to Leuconostoc OTU4482944 (Pearsons *r* = 0.893) and a Lactococcus OTU1100972 (Pearsons *r* = 0.775), which are both known members of the probiotic Order Lactobacillales. Also found in this cluster were chlorogenic acids and feruloyl-tyramine like compounds. These compounds were much more abundant in the early days of the fermentation, and highly glycosylated forms of ferulic acid were rapidly depleted through time.

## 4. Discussion

Home food fermentation is seeing a resurgent interest in many western societies due to the potential for consumption of live probiotic organisms [19,20]. The foods selected for this study were grown organically to determine the natural process occurring in an ‘in-home’ fermented food, without any known preservatives or other non-biological chemical additives. We applied modern mass spectrometry and microbiome bioinformatics approaches to provide a global overview of the changes occurring in the fermented foods and an untargeted assessment of specific microbial species and metabolite dynamics. Molecular networking and MS/MS similarity searching applied to fermented food studies revealed metabolite changes from knowns and unknowns without any *a priori* biased targeting of particular molecular groups [21,22].

Principle coordinate analysis plots of metabolome dissimilarity enabled visualization of the changes through time compared to their autoclaved sterile control samples. The chemistry of all four foods was significantly different from controls throughout the fermentation, except on the first day which was sampled prior to incubation. The beta-diversity plots also showed that the foods were highly different from each other as expected, but also that the degree of change in the foods through time depended on the vegetable. Carrots, radishes and peppers all had strong progressive changes through PCoA space reflecting a dynamic metabolome through the entire fermentation process, but beets changed more rapidly within the first two days and then little movement was observed through PCoA space in the final three days of fermentation. This indicates that the rate of natural food fermentation depends on the vegetable and desired flavoring or nutritive value may also depend on timing of consumption of fermentation arrest. Although a four-day fermentation time is common for ‘in-home’ fermented food recipes, other vegetable fermentation procedures often employ longer ferment times than that used here. In a kimchi fermentation, for example, similar dynamic changes were observed in the first 5 to 10 days of fermentation, followed by a shift to a more static state at 20-30 days and then a final maturation stage at 50 days [7]. Here, the molecular diversity of the foods peaked at three days (two days for peppers) and then all foods had a slight decrease in metabolite diversity in the final day, indicating that the microbial process had slowed. If attaining the most diverse metabolite repertoire of a fermented food is a desired flavor or nutritive point, it may be useful to determine when this peak occurs within each home fermentation recipe and food type. An RF regression analysis of the metabolome changes through time also indicated that the process had begun to slow after day three as the machine-learning based predictions were not able to differentiate day three from day four in all foods. This untargeted approach to metabolomic profiling of fermented foods provides unique insight into the overall metabolite changes associated with ‘in home’ salt-based fermentations. Microbial growth during natural fermentation of brined vegetables has been described to occur in four stages: initiation, primary fermentation, secondary fermentation, and post-fermentation spoilage [23]. We saw a similar trend here with an initial delay in metabolome changes followed by a period of rapid chemical transformation and a final day of relative stasis.

The microbiome profiles of the fermented foods were somewhat surprising. Probiotic lactobacilli did not come to dominate the fermentation as expected, instead, members of the Enterobacteriaceae took over as the fermentation progressed. Members of the Lactobacillales (lactic acid bacteria) were also abundant through the fermentation process, but these were primarily *Leuconostoc* and *Lactococcus* spp., who are not commonly administered as probiotic organisms. Leuconostocaceae are common constituents of naturally fermented vegetables such as kimchi and can often dominate these microbial fermentations (8). The probiotic *Lactobacillus brevis* [24] was detected in both beets and peppers but was of low relative abundance (<5%). A high abundance of Enterobacteriaceae spp. is not uncommon in fermented food microbiome profiles, for example, the early days of carrot and cabbage (sauerkraut) fermentation had an abundance of these organisms whereas lactic acid bacteria came to dominate at later stages of the fermentation [25,26]. Erwinia was a predominant fermenting organism in all four vegetables in this study. *E. amylovora* is a common plant pathogen known to infect a variety of different plant species. The presence of this bacterium during the fermentation may reflect its ability to invade plant tissues if compromised, possibly due to chopping prior to the fermentation. It is also a robust fermenter of sugars and has been explored industrially for its production of desirable microbial products [27] and has been identified in alcoholic fermentations of different plant parts, such as pulque (agave) [28]. Prior to the fermentation the microbiome of the vegetables in this study were dominated by plant-associated pseudomonads, which were quickly depleted after day 1 or 2 when the Enterobacteriaceae started to dominate. In peppers and radishes, the primary Enterobactericeae member dominating the fermentation was also present on the vegetable prior to the fermentation process, in contrast, carrots and beets had low to undetectable amounts of these organisms at time zero. This is interesting in light of recent characterizations of the vegetable skin microbiome which have shown that different species of these edible plants have unique microbiomes and this beta-diversity is driven by the relative abundances of Enterobacteriaceae [29]. The dominance of this family indicates that organic food fermentation may actually reduce the selection for probiotic organisms such as lactobacilli that are generally regarded as beneficial to the human gut microbiome. Spontaneous fermentations are known to be more challenging to control and many industrialized fermented food producers use starter cultures to directly manipulate fermentation outcomes [30]. Future work comparing organic and non-organic food fermentations are needed to determine if this microbial property is unique to organically fermented foods or of these vegetables in particular.

Another aspect of the perceived health benefits of whole foods are plant natural products such as flavonoids, anthocyanins and triterpenoids. These compounds have been shown to have a variety of health benefits and consumption of whole foods is a significant dietary source of these compounds [31,32,33,34]. Furthermore, the gut microbiome is known to degrade the glycoside forms of these compounds producing aglycones that can interact with the gut epithelium and potentially increase their health benefits [35,36]. As such, we investigated the production of these plant products in the organically fermented foods through time and found, similarly to the microbial profiles, that the production of these compounds depended on the vegetable. In beets, peppers and radishes, many of these natural products increased during the fermentation, including their aglycone forms. In carrots, however, we observed a reduction in the abundance of specific flavonoids and triterpenoids as the process progressed. Anthocyanins, complex flavonoid natural products, were particularly abundant in radishes and showed a progressive increase in their detection through the fermentation. A *Lactococcus* sp. and *Leuconostoc* sp. were highly correlated to their production in radishes. Although one cannot infer that they are produced by these organisms, this association may further implicate probiotic mechanisms that exist within the Lactobacillaceae, by releasing plant natural products during fermentation. Thus, natural fermentation of vegetables increases the production of specific plant natural products with demonstrated health benefits, but this is dependent on the vegetable consumed. The microbial diversity observed between the different vegetables fermented in this study may have a direct influence on the nutritive value of the fermented food.

## 5. Conclusions

In summary, ‘in-home’ salt-based fermentation of organic vegetables induced drastic changes in the microbiology and metabolite profiles of these foods. Our results indicate that the presumed nutritive and probiotic value of this process is highly dependent on the vegetable and microbiome that comes to dominate the process. Release of beneficial plant natural products into the brine better enabling their consumption may be a lesser appreciated mechanism behind the nutritive value of fermented food consumption, particularly in radishes and peppers. However, further studies are needed to determine whether fermentation of ‘organic’ vegetables preferentially selects for Enterobacteriaceae and if this is different from conventionally processed vegetables available to consumers. A deeper look into the microbial process occurring in ‘in-home’ fermented foods is needed to better understand how to optimize their health benefits. Starter cultures, such as lactobacilli which may have inherent probiotic properties, have been shown to enable a more controlled fermentation and may greatly enhance the nutritive value of the fermented food process [28]. Experimentation with this starter cultures on different vegetables in an ‘in-home’ setting may lead to better guidelines on which foods should be selected for home fermentation and how to optimize the process to maximize health benefits.

## Supplementary Materials

The following are available online at https://www.mdpi.com/2218-1989/9/8/165/s1: Figure S1: experimental design, Figure S2: Metabolome PCoA, Figure S3: pH changes, Figure S4-S7: Microbiome data with taxa assignments. Figure S8: Plant natural product network by vegetable. Figure S9: Metabolite annotation mirror plots. Table S1: Variation explained by RF regressions. Table S2: Pearson corrleations of metabolite richness and time.
