# Supplementary material for: Microbial Transformations of Organically Fermented Foods
Source: Metabolites. 2019 Aug 10;9(8):165. doi: 10.3390/metabo9080165 (PMC6724132; doi:10.3390/metabo9080165)
Supplement: Supplementary file 1 [file metabolites-09-00165-s001.pdf]

Microbial Transformations of Organically Fermented Foods

Ruma Raghuvanshi[1], Alyssa G. Grayson[1], Bella Schena[1], Onyebuchi Amanze[1], Kezia Suwintono[1] and Robert A. Quinn[1]

[1]Department of Biochemistry and Molecular Biology, Michigan State University, East Lansing, MI

Supplemental Data.

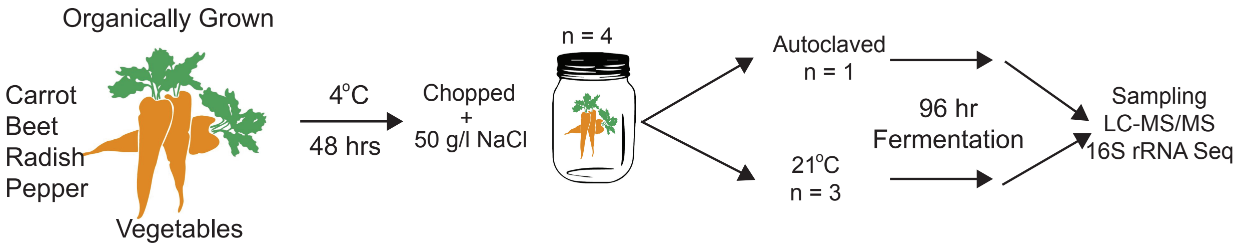

Fig. S1. Experimental design schematic.

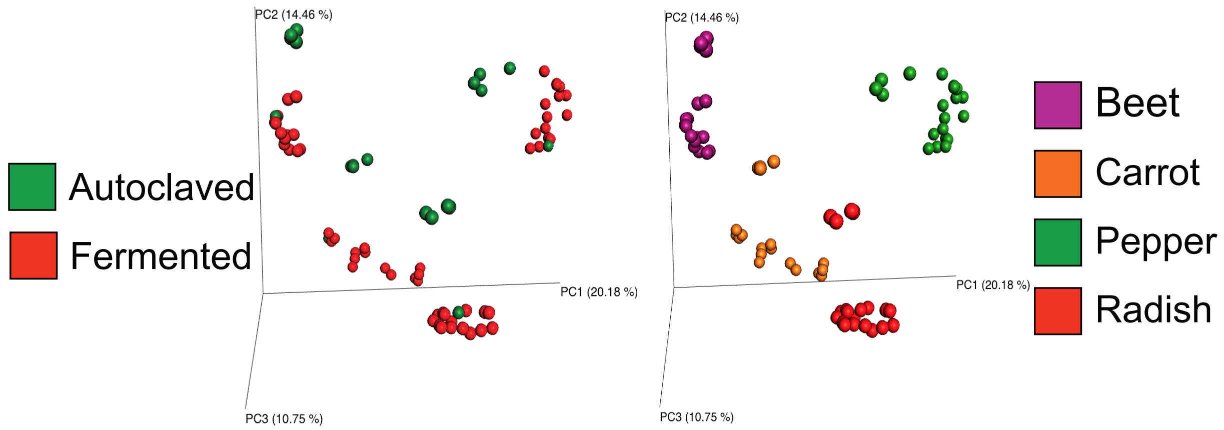

Fig. S2. Principle coordinate plots of the Bray-Curtis distances of the metabolomic data of autoclaved and fermented vegetables colored by their sterilization treatment and vegetable type.

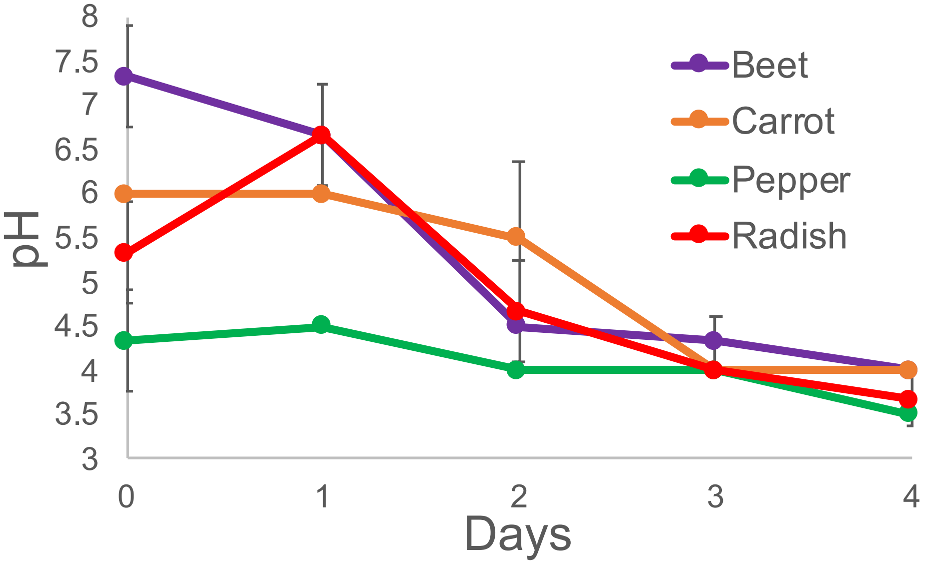

Fig. S3. pH changes in the brine of each vegetable during fermentation.

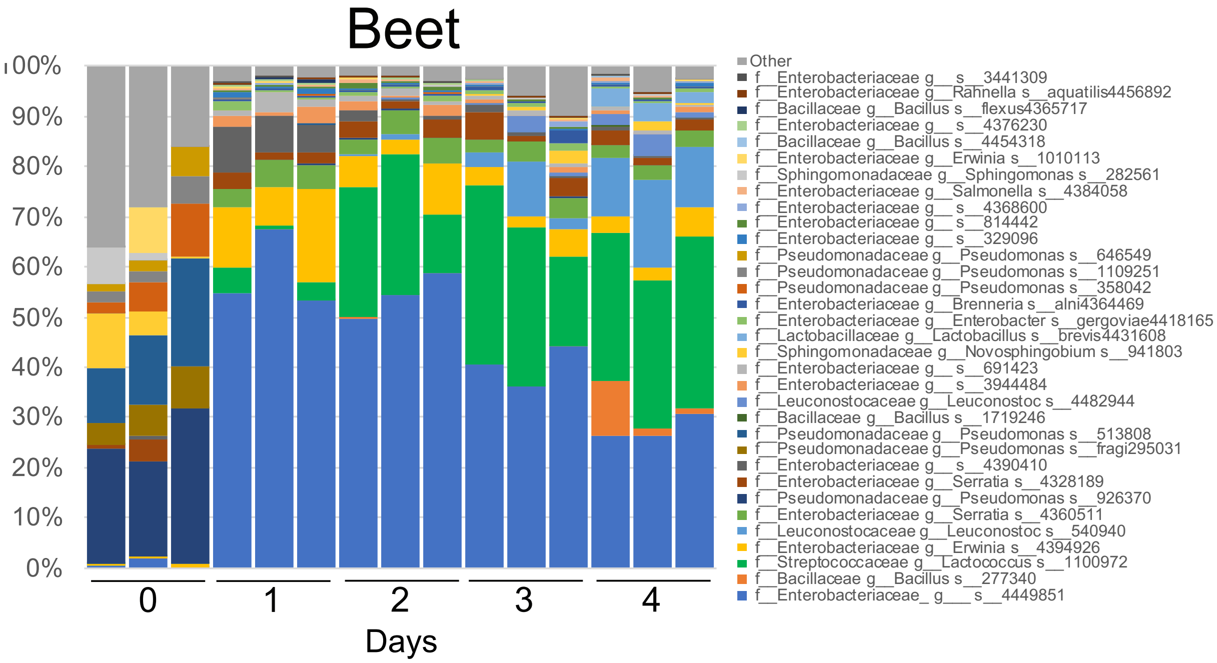

Fig. S4. Beet microbiome changes according to the 16S rRNA gene microbiome sequencing and assignment of taxonomy with the OTU clustering method. The three replicates are shown individually.

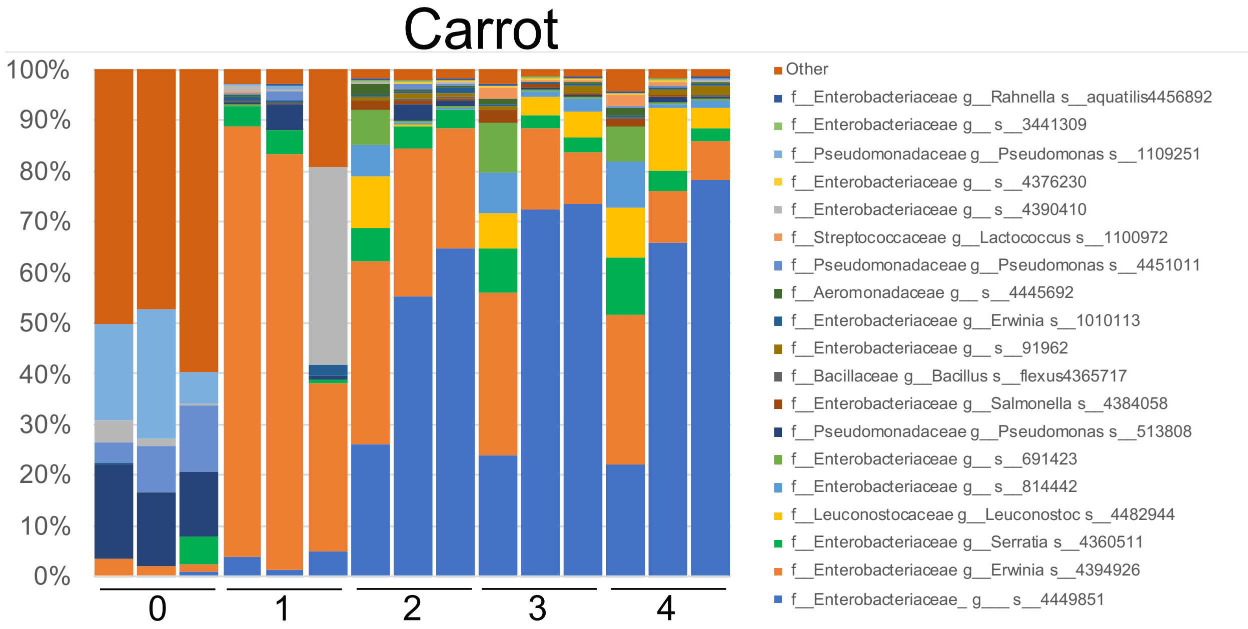

Fig. S5. Carrot microbiome changes according to the 16S rRNA gene microbiome sequencing and assignment of taxonomy with the OTU clustering method. The three replicates are shown individually.

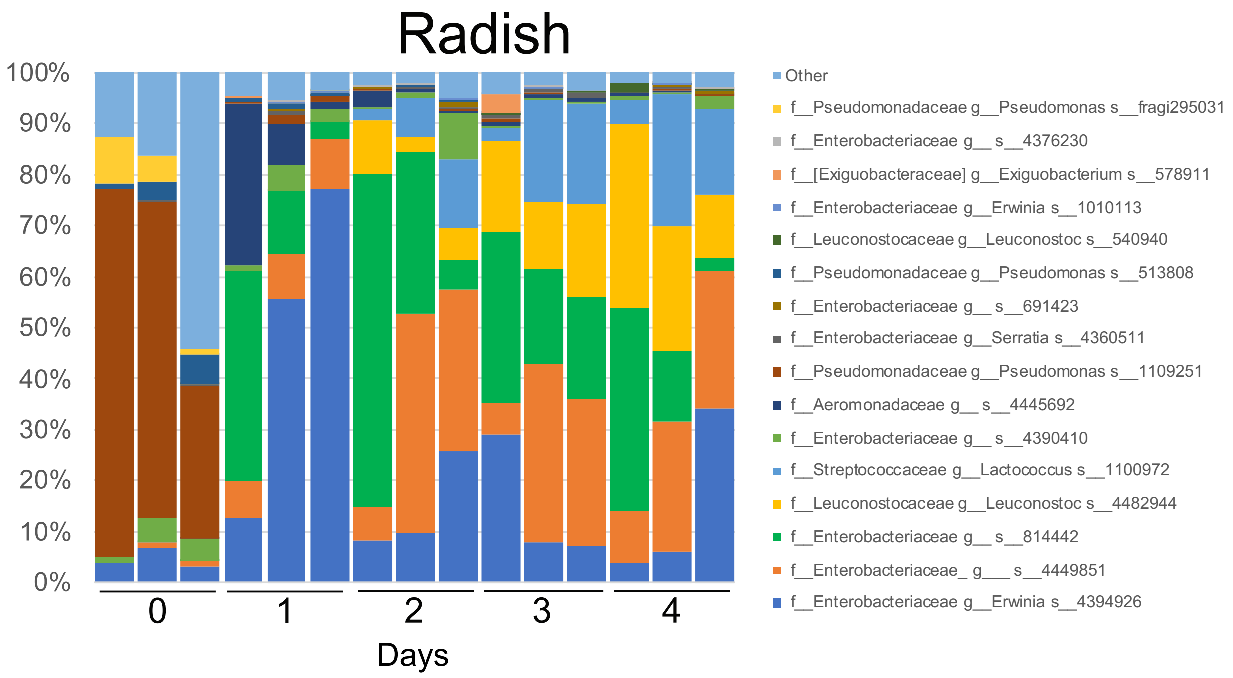

Fig. S6. Radish microbiome changes according to the 16S rRNA gene microbiome sequencing and assignment of taxonomy with the OTU clustering method. The three replicates are shown individually.

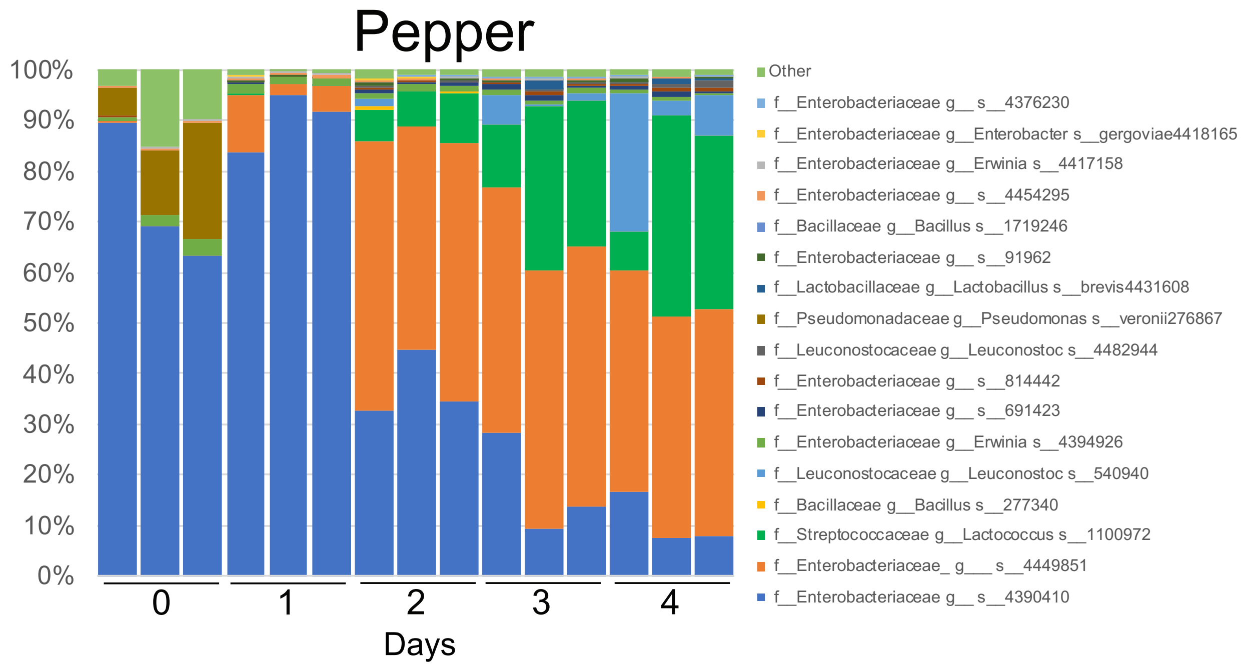

Fig. S7. Pepper microbiome changes according to the 16S rRNA gene microbiome sequencing and assignment of taxonomy with the OTU clustering method. The three replicates are shown individually.

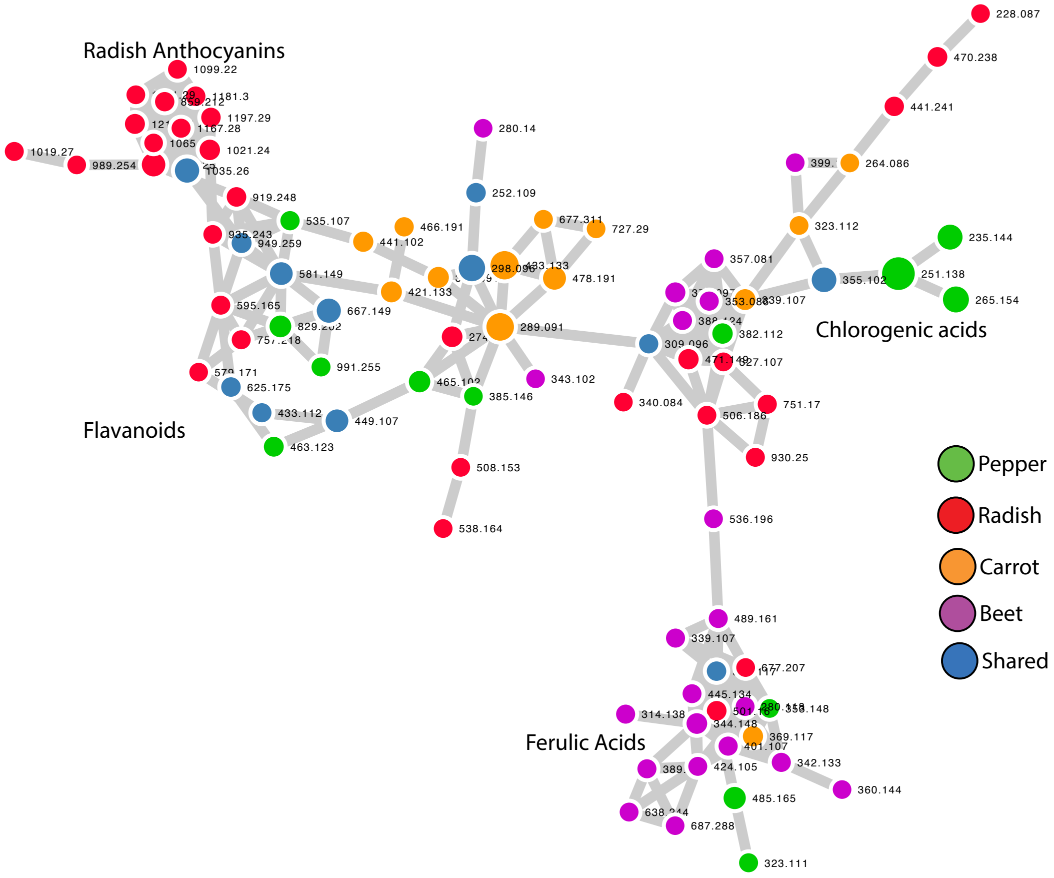

Fig. S8. Plant natural product and anthocyanin molecular network colored according to vegetable source. The molecular network cluster topology is the same orientation as in Figure 5.

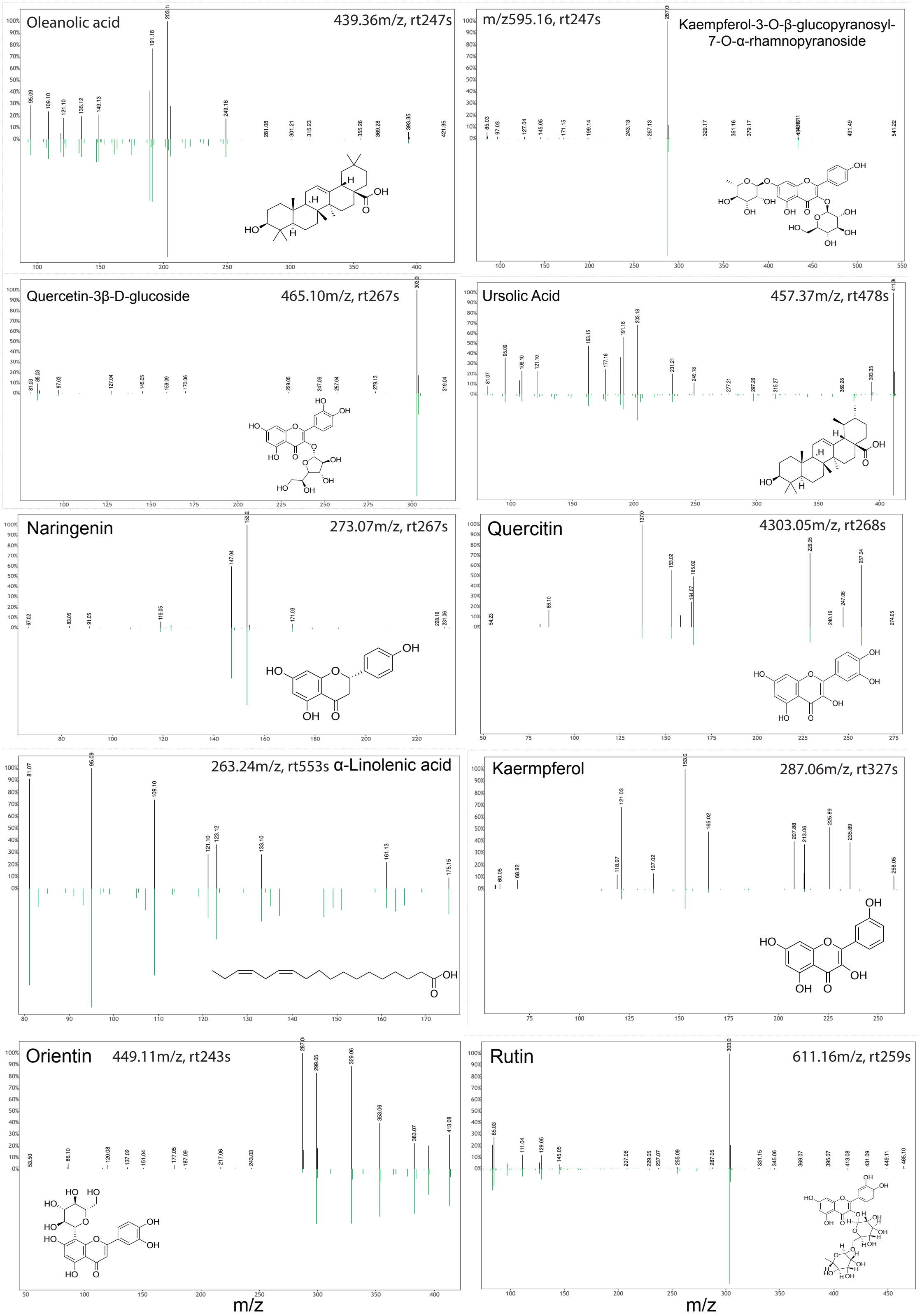

Fig. S9. Mirror plots of unknown spectra with library spectra showing identification of compounds at the metabolomics standards initiative level 2. The query spectrum is on the top and the library spectrum on the bottom.

Supplemental Tables.

Table S1. Percent of variance explained from RF regression of metabolomic data and time in days for the autoclaved and conventionally fermented vegetables.

	Autoclaved	Conventional
Beet	-33.10	83.55
Carrot	-55.87	67.98
Pepper	-24.91	78.62
Radish	-61.3	67.98

Table S2. Pearson correlations and p-values of significance for the correlation of metabolite richness and fermentation time in days for each vegetable.

	Beet	Carrot	Pepper	Radish
Pearson	0.869	0.780	-0.223	0.637
p-value	0.00026	0.00059	0.43	0.011
